# Electroencephalography-based endogenous brain–computer interface for online communication with a completely locked-in patient

**DOI:** 10.1186/s12984-019-0493-0

**Published:** 2019-01-30

**Authors:** Chang-Hee Han, Yong-Wook Kim, Do Yeon Kim, Seung Hyun Kim, Zoran Nenadic, Chang-Hwan Im

**Affiliations:** 1 Department of Biomedical Engineering, Hanyang University, Seoul, 04763 South Korea; 2 Department of Neurology, College of Medicine, Hanyang University, Seoul, 04763 South Korea; 3 Department of Biomedical Engineering, University of California, Irvine, CA 92697 USA

**Keywords:** Brain-computer interface (BCI), Completely locked-in state (CLIS), Electroencephalography (EEG), Pattern classification, Riemannian geometry

## Abstract

**Background:**

Brain–computer interfaces (BCIs) have demonstrated the potential to provide paralyzed individuals with new means of communication, but an electroencephalography (EEG)-based endogenous BCI has never been successfully used for communication with a patient in a completely locked-in state (CLIS).

**Methods:**

In this study, we investigated the possibility of using an EEG-based endogenous BCI paradigm for online binary communication by a patient in CLIS. A female patient in CLIS participated in this study. She had not communicated even with her family for more than one year with complete loss of motor function. Offline and online experiments were conducted to validate the feasibility of the proposed BCI system. In the offline experiment, we determined the best combination of mental tasks and the optimal classification strategy leading to the best performance. In the online experiment, we investigated whether our BCI system could be potentially used for real-time communication with the patient.

**Results:**

An online classification accuracy of 87.5% was achieved when Riemannian geometry-based classification was applied to real-time EEG data recorded while the patient was performing one of two mental-imagery tasks for 5 s.

**Conclusions:**

Our results suggest that an EEG-based endogenous BCI has the potential to be used for online communication with a patient in CLIS.

## Background

Brain–computer interface (BCI) is an emerging technology capable of translating human intentions into control signals, thereby enabling people to communicate with their external environment without any kinesthetic movement [1]. BCI technology has primarily targeted patients with severe or complete motor dysfunction due to various neurological disorders and cardiovascular diseases. Among these target groups, patients with amyotrophic lateral sclerosis (ALS) have the potential to benefit most from BCI technology because they generally maintain unimpaired cognition even after complete loss of voluntary motor function. ALS is characterized by a rapidly progressive degeneration of motor neurons, leading to muscle weakness, respiratory paralysis, and ultimately death [2]. Therefore, patients in advanced stage of ALS usually experience a physical condition, referred to as a locked-in state (LIS) [3], in which they remain aware of their external environment but lose control of voluntary muscles except for control over eye movements [4]. In the final stages of ALS, the patients even lose the oculomotor function and are isolated from their external environment owing to the lack of communication means [5]. This stage is referred to as the completely locked-in state (CLIS).

Various brain-imaging modalities have been used to implement BCI systems with the ultimate goal of communicating with patients in LIS. Among these, electroencephalography (EEG) has been the most widely used modality because of its portability, non-invasiveness, high temporal resolution, and a reasonable cost compared to other neuroimaging tools, such as near-infrared spectroscopy (NIRS), functional magnetic-resonance imaging (fMRI), and magneto-encephalography (MEG) [6]. Over the last couple of decades, EEG-based BCI systems have been developed with elaborately designed paradigms [7–12], with greatly promising practical applications. For example, steady-state visual evoked potential (SSVEP) [7, 8], event-related potential (ERP) [9, 10], and motor imagery (MI) [11, 12] are three major EEG-based BCI paradigms. These can be roughly classified into exogenous and endogenous BCI paradigms. The SSVEP and P300 BCIs are representatives of exogenous BCI paradigms. They use external stimuli such as flickering LEDs or auditory beeps to evoke discriminative brain patterns. On the other hand, MI-based systems represent endogenous BCIs as they do not use any external stimuli. If a user of an endogenous BCI performs one of the designated mental tasks, the system will recognize the specific task from self-regulated EEG patterns. The absence of external stimuli is especially advantageous for the patients in CLIS because of their complete lack of motor function. In addition, endogenous BCIs are more straightforward, more convenient, and may be faster than exogenous BCIs. Over the past decade, most EEG-based BCI systems were tested with healthy individuals [13, 14]. Recently, the number of EEG-based BCI studies in patients with ALS has steadily increased, several of which have yielded promising results [4, 5, 15–25]. Unfortunately, EEG-based BCI systems for online communication with patients in CLIS have been mostly unsuccessful. A recent study demonstrated the potential of using a vibrotactile stimulation-based exogenous BCI paradigm for communication with patients in CLIS [26]. However, no study has yet reported on the successful online communication with CLIS patients using EEG-based endogenous BCI systems, which do not require external stimuli, but instead use neural signals volitionally modulated by BCI users. To the best of our knowledge, there were only two EEG-based endogenous BCI studies with CLIS patients; however, both failed to establish online communication [21, 27].

In this study, we developed an EEG-based online BCI system for the classification of different mental-imagery tasks conducted by a patient in CLIS. An endogenous BCI paradigm based on mental-imagery tasks was designed, and a single patient in CLIS with advanced stage of ALS participated in this study. Offline and online experiments were conducted to validate whether the proposed BCI could be used for real-time communication with the patient. The test–retest reliability of our BCI system was also investigated using training datasets obtained on different days.

## Methods

### Participant characteristics

A 62-year-old woman with severe ALS was recruited for this study. In November 2012, she was diagnosed with clinically probable ALS according to the Airlie House diagnostic criteria [28]. After diagnosis, her motor weakness rapidly progressed and propagated to all motor subsystems, including bulbar and respiratory functions. In late 2013, mechanical ventilator and transcutaneous endoscopic gastrostomy were needed. Subsequently, all voluntary motor functions including ocular movement and eye blinking were completely lost as of early 2015. Despite of the complete loss of voluntary movement including eye movement, the participant’s event-related potential (ERP) waveform characteristics suggested that her hearing and cognitive functions were preserved. The participant’s CLIS diagnosis was additionally supported by the discovery of meaningful tearing after listening to sorrowful news of her family member’s death. Her ALSFRS-R was zero [29] and no alternative means of communication with the patient were available at the time of this study. The patient’s husband provided written informed consent prior to all experiments. This study was reviewed and approved by the Institutional Review Board Committee of Hanyang University Hospital (HYUH 2015–11–031-001) and conformed to the tenets of the Declaration of Helsinki.

### Experimental protocols

All experiments were conducted in a hospital setting where the patient had stayed for 2 years. We visited her four times on different days (first visit: 28th Jan 2016; second visit: 4th Feb 2016; third visit: 12th Aug 2016; and last visit: 9th Dec 2016) and performed four different experiments at each visit. All the experiments were performed between 10 am and 12 pm because the patient’s husband told us that she had usually been in a good condition in the morning. During the experiments, all instructions were directly presented to the participant using noise-cancelling headphones.

**Fig. 1:**
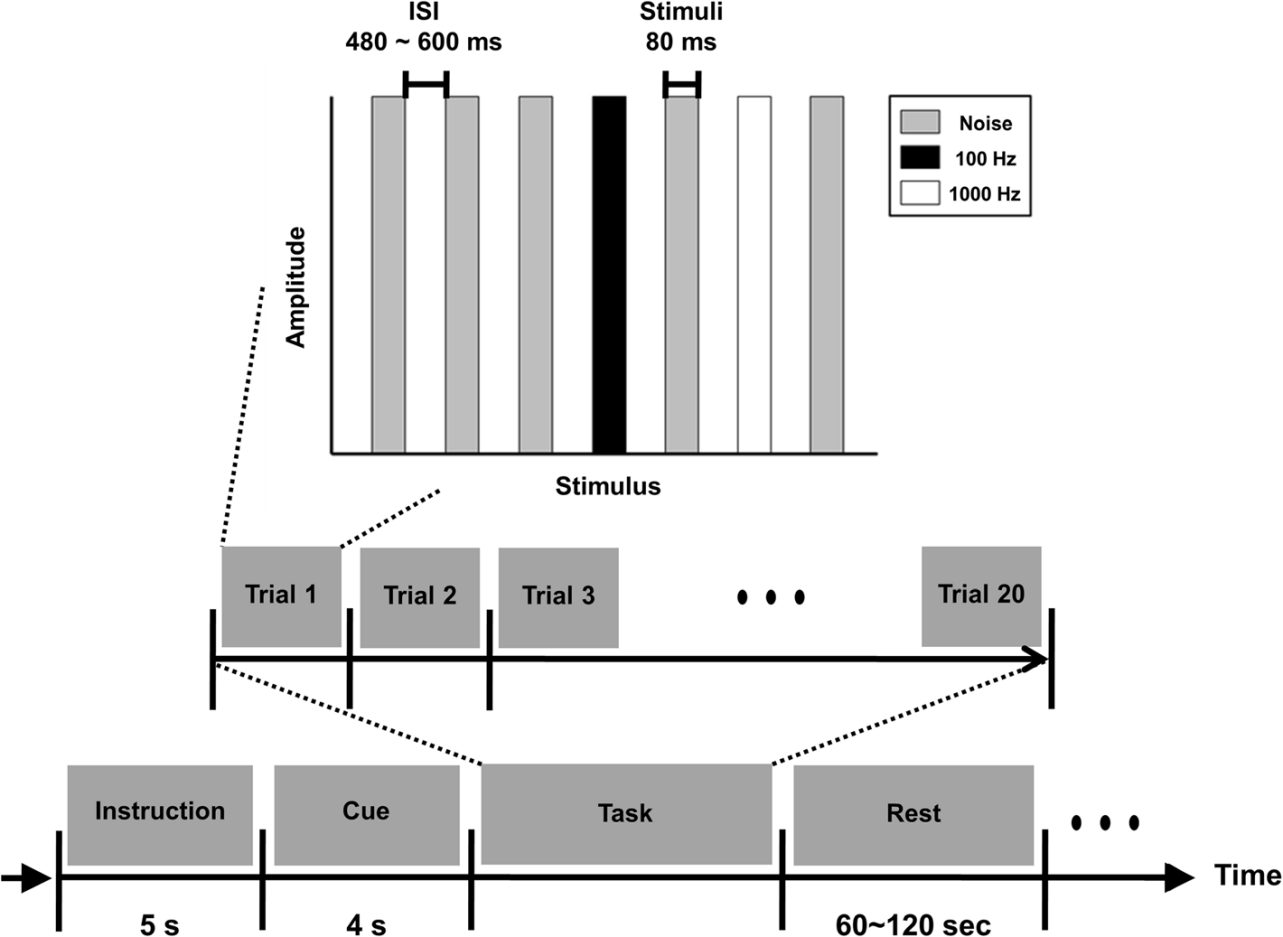
Schematic diagram of the auditory oddball offline paradigm

On the first and last visits, we assessed whether the patient’s alertness was high enough to conduct the EEG-based BCI experiments. Her auditory and cognitive functions were evaluated based on auditory ERPs. An auditory oddball paradigm with a cognitive task was used for this test (see Fig. 1). At the beginning of a run, a start message was presented for 5 s, and we instructed the subject to prepare for a given test during this period. After the start message, a brief instruction to attend to a left-high or right-low beep was presented for 4 s. We asked the subject to perform trials corresponding to the instruction. A single trial consisted of three different noise sounds, a high-frequency (1000 Hz) beep, and a low-frequency (100 Hz) beep. The inter-stimulus interval (ISI) ranged from 480 to 600 ms, and each stimulus was presented for 80 ms. Note that the high- or low-frequency beeps were presented through the left and right headphone, respectively. There were 20 trials in a run, and the patient completed a total of 6 runs. After confirming that the subject’s auditory and cognitive functions were not completely impaired, offline and online experiments based on mental-imagery tasks were conducted during the second and third visits, respectively.

**Fig. 2:**
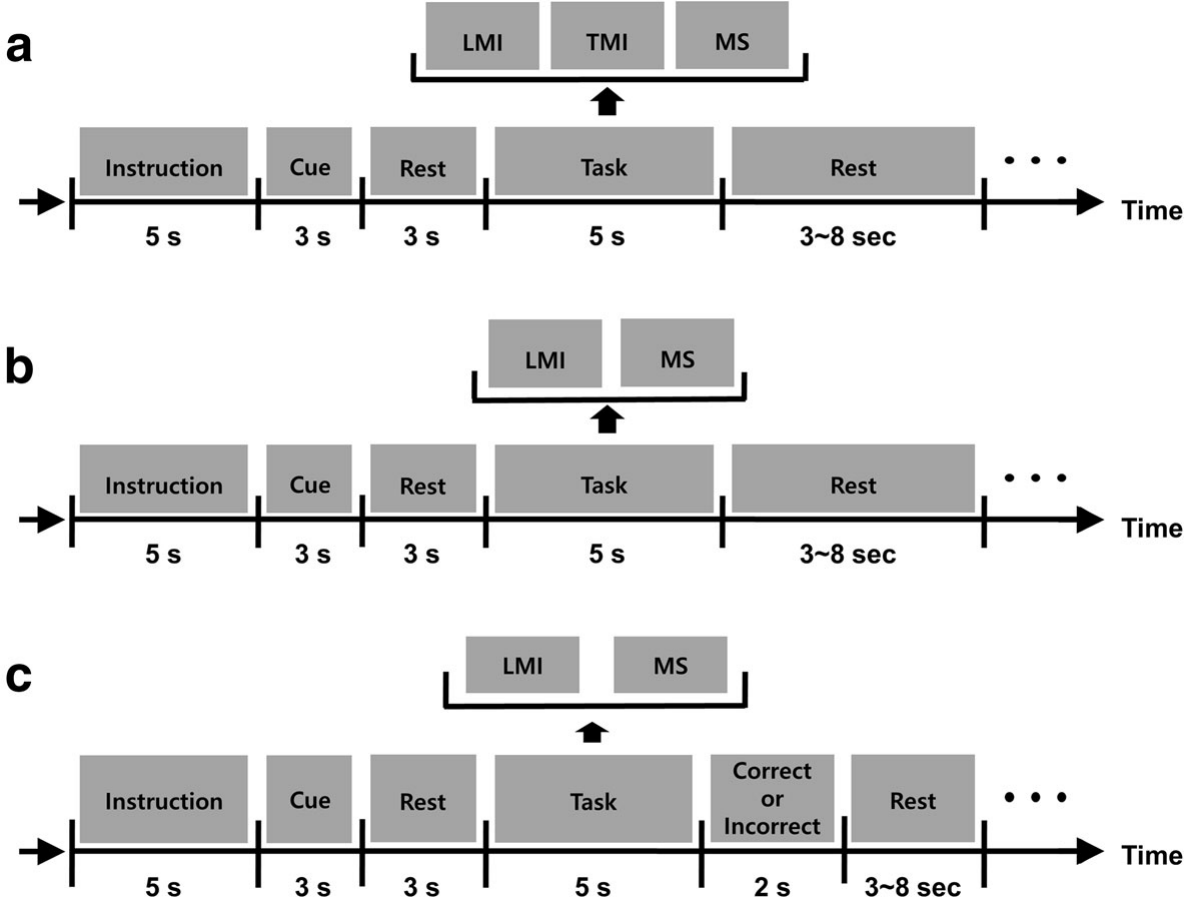
**a** The offline experimental paradigm. **b** The training paradigm of the online experiment. **c** The test paradigm of the online experiment. LMI, TMI, and MS indicate left-hand motor imagery, tongue motor imagery, and mental subtraction, respectively. The offline experiment and the training paradigm of the online experiment were similar except for the number of tasks. In the test paradigm of the online experiment, auditory feedback was provided in accordance with the online results of the participant’s EEG data analysis

For the offline experiment based on mental-imagery tasks, we designed three different mental tasks: left motor imagery (LMI), mental subtraction (MS), and tongue motor imagery (TMI). During LMI and TMI, the patient was asked to perform kinesthetic imaginations of left-hand and tongue movements, respectively. During MS, the patient was asked to sequentially subtract a small number (e.g. 7) from a three-digit number (e.g. 428) as fast as she could (e.g. 428–7 = 421, 421–7 = 414, 414–7 = 407, …). The pairs of numbers used were not repeated to prevent the patient from becoming accustomed to the problem. We tested both motor and non-motor imagery tasks because some previous studies demonstrated that the combination of motor and non-motor imagery tasks can enhance the overall classification accuracy of binary classification BCI [30, 31]. Figure 2a provides the schematic diagrams of the offline experiment. Instructions were presented for 5 s to provide the participant with a short preparation time before starting each experiment. At the beginning of each trial, a variable rest period (3–8 s) was provided, and then the auditory cue (verbal instructions generated by a computer) for a specific mental task was presented to the subject for 6 s (the cue was presented in the first 3 s). After presentation of a pure-tone beep, the patient performed the given mental task for 5 s. A single experimental run was composed of six independent trials; thus, each mental task was performed twice in a random order during each run. The patient performed 10 runs in total, consequently performing each task 20 times.

In the online experiment based on mental-imagery tasks, the pair of mental tasks that showed the highest classification performance in the offline analysis, i.e. the combination of LMI and MS, was selected. Figure 2b and c are schematic diagrams of the training and test paradigms of the online experiment, respectively. The subject participated in 10 training runs without any feedback before the main online test experiment. Each training run was composed of four random, counter-balanced executions of either LMI or MS. The participant then completed four online test runs right after the training runs were complete. Each test run was composed of 10 trials (5 trials for each task). Real-time auditory feedback was provided immediately after each trial based on the classification results (please watch the supplementary video at https://youtu.be/zQWtSQOV50Q). The subject was able to recognize whether the online results were correct or not through auditory feedback.

### EEG data acquisition

EEG data were recorded using a multi-channel EEG acquisition system (ActiveTwo, BioSemi, Amsterdam, The Netherlands). For the offline and online experiments, 19 EEG electrodes (Oz, O1, O2, Cz, C3, C4, T7, T8, Fz, FC1, FC2, FC5, FC6, F3, F4, F7, F8, AF3, and AF4) were used. The use of electrodes in the posterior region was avoided because the patient was in a supine position on a bed during experiments. The ground electrode was replaced with two electrodes, a common-mode-sense (CMS) active electrode and a driven-right-leg (DRL) passive electrode, both of which were located in the central region (near CP1 and CP2). The offset voltage between the A/D box and the body was maintained between 25 and 50 mV, as recommended by the EEG device manufacturer. The EEG data were sampled at 2048 Hz.

### Analysis of ERPs by auditory oddball test

Raw EEG data acquired via the auditory oddball paradigm were pre-processed to remove unwanted artefacts using MATLAB software (MathWorks, Natick, MA, USA). A pre-processing algorithm used in a previous ERP study [32] was adopted. The raw EEG data were re-referenced with a common average reference (CAR) that uses the mean of all electrodes as a reference. The DC-level components in all channels were removed by subtracting the mean of the time series for each channel, and the EEG data were band-pass filtered at 1- and 30-Hz cut-offs using a third-order Butterworth zero-phase filter. The pre-processed EEG data were epoched from 100 ms pre-stimulus to 1000 ms post-stimulus. The averaged value of the pre-stimulus interval was subtracted from the selected epochs for baseline correction. We assessed whether the remaining epochs contained significant physiological or environmental artefacts (amplitude exceeding ±75 μV). Because no epoch satisfied this condition, all epochs were used in subsequent analyses.

Mismatch negativity (MMN), the negative peak generated near 200 ms where a human can hear some specific sounds [33, 34], was confirmed to determine whether the patient could hear all instructions presented through the headphones. In this study, the MMN was defined by a minimum amplitude between 100 and 300 ms post-stimulus. There were three different stimuli in our auditory oddball test: white noises (600 times), high-tone beeps (120 times), and low-tone beeps (120 times). In the MMN analysis, the noises and beeps were regarded as standard and deviant stimuli, respectively. The ratio of noises to beeps was 2.5:1. Two hundred forty epochs were randomly selected from 600 noise trials and were compared with the 240 beep epochs. P300 components in ERPs were evaluated on the midline electrodes (Fz, Cz, and CPz) as well as the bilateral temporal lobe electrodes (T7 and T8) to confirm the patient’s cognition. In the ERP analysis, high and low-tone beeps were used as target and non-target stimuli, respectively. A statistical analysis was performed to confirm the significance of the MMN and P300 components. For MMN, each epoch from 100 ms to 300 ms in deviant and standard trials was segmented using a moving window of 62.5 ms with 50% overlap for each channel. For P300, each epoch from 200 ms to 700 ms in all target and non-target trials was segmented using a moving window of 62.5 ms with 50% overlap for each channel. Then, paired t-tests were performed for all pairs of corresponding time segments to find time intervals with a significant difference (*p* < 0.05) between the target and non-target conditions. The *p*-values of all pairs were corrected using the Bonferroni correction method for multiple comparisons.

### Event-related (de) synchronization analysis

The event-related desynchronization (ERD) and synchronization (ERS) patterns were evaluated for each mental task to validate whether the patient actually conducted the given mental tasks. The raw EEG data for each mental task were filtered using CAR and band-pass filtered at 4 to 45 Hz. Then, each 6-s epoch from − 1 to 5 s was extracted from the filtered EEG signals and was down-sampled from 2048 Hz to 512 Hz. The event-related spectral perturbation (ERSP) was calculated using the function newtimef in the EEGLAB toolbox (https://sccn.ucsd.edu/eeglab/index.php). The function returns estimates of the ERSP across event-related trials for each channel times in the series. As already mentioned, the EEG data from − 1 to 0 s was used as the baseline for the ERSP evaluation. The target frequency bands for each mental task were set differently: alpha (8–13 Hz) and low-beta (13–20 Hz) for LMI and TMI; theta (4–7 Hz) and alpha (8–13 Hz) for MS. The ERSP patterns at different target electrodes were also observed for each mental task: C3 and C4 for LMI and TMI; AF3 and AF4 for MS. Topographical distributions were drawn using the averaged ERSP values from 0 to 5 s for each frequency band.

### Analysis of EEG data by the BCI paradigm based on mental-imagery tasks

Raw EEG data obtained during the mental task paradigm were pre-processed using MATLAB software. The raw EEG signals were spatially filtered using a CAR to compensate for common noise components. DC components were removed by subtracting the mean of the time series for each channel, and the EEG data were then band-pass filtered at 4- and 45-Hz cutoffs using a fifth-order Butterworth zero-phase filter. For the pattern classification of different mental tasks, each 5-s epoch (0–5 s after task onset) of all trials for each task was extracted from the pre-processed EEG signals. Finally, each epoch was down-sampled from 2048 Hz to 512 Hz.

The performance of two different classification frameworks were evaluated by offline analysis. The first framework uses spatial covariance matrices as EEG descriptors and depends on Riemannian geometry (RG) to classify the matrices for each class. A classification framework by RG was selected and applied to our EEG dataset because it showed excellent classification performance compared to other methods using spatial covariance matrices as the EEG signature [35–37]. In 2016, Barachant et al. [35] proposed two methods based on RG that directly use the covariance matrices for classification and have shown that their RG-based frameworks could outperform a reference method. In the next year, they also introduced several new kernels based on RG for classifying covariance matrices. An approach using one of their developed kernels significantly outperformed a reference method in various BCI paradigms. The framework first extracts spatial covariance matrices (SCMs) for each class [35]:

$$ {\mathbf{P}}_i=\frac{1}{N_t-1}{\mathbf{X}}_i{{\mathbf{X}}_i}^T $$

where **P**_*i*_ represents an SCM for the *i*-th trial of each class. **X** and *N*_*t*_ are a short-time segment of EEG signals and the number of samples in a trial, respectively. Because SCMs are symmetric positive-definite (SPD) matrices belonging to the manifold *P*(n), the distance between any two SPD matrices **P**_1_ and **P**_2_ is defined using Riemannian geometry, as follows:

$$ {\delta}_R\left({\mathbf{P}}_1,{\mathbf{P}}_2\right)={\left[\sum \limits_{i=1}^n\log {}^2{\uplambda}_i\right]}^{1/2} $$

where λ_*i*_ (*i* = 1, …, *n*) are the eigenvalues of **P**_1_^−1^**P**_2._ When we have a set of *M* covariance matrices, the Riemannian mean can be computed as

$$ \upvartheta \left({\mathbf{P}}_1,\dots, {\mathbf{P}}_M\right)=\mathit{\arg}\underset{\mathbf{P}\in P(C)}{\min}\sum \limits_{m=1}^M{\delta}_R^2\left(\mathbf{P},{\mathbf{P}}_m\right) $$

The Riemannian means are then mapped onto the Riemannian tangent space for classification. This mapping method is called tangent space mapping [35]. Through this mapping method, the matrices can be vectorized and handled as Euclidean objects. This mapping method allows the use of more advanced classifiers within the Riemannian space and returns a new feature set. The size (**S**) of the new feature set is defined as follows:

$$ \mathbf{S}={\mathbf{N}}_{\mathrm{elec}}\ast \left({\mathbf{N}}_{\mathrm{elec}}+1\right)/2 $$

where **N**_elec_ represents the number of channels, which was 19 in this study. As a result, the feature vectors of training and test sets in our online experiment were matrices of 190 × 40 (20 trials for each class in a training session) and 190 × 40 (20 trials for each class in four test sessions), respectively. A procedure for selection of variables is then applied in order to decrease the space’s dimensionality. A one-way ANOVA is used to select the most discriminant variables. Finally, linear discriminant analysis (LDA) was selected as a classifier and performed to each pair of class. The leave-one-out cross validation (LOOCV) method in offline analysis was applied to calculate the classification accuracy owing to the relatively small number of task trials (20 trials per task). A MATLAB toolbox, the Covariance Toolbox, was used (https://github.com/alexandrebarachant/covariancetoolbox) for these analyses.

The second classification framework was a traditional one using spectral band powers and their inter-hemispheric asymmetry ratios as EEG features. The frequency bands were separated into five sub-frequency bands: theta (4–7 Hz), alpha (8–13 Hz), low beta (14–20 Hz), high beta (21–30 Hz), and gamma (31–45 Hz). In order to extract the power spectral density (PSD) features, each epoch was divided into 1-s segments with 50% overlap, resulting in a total of nine-time segments. Each segment was then transformed into the frequency domain using a fast Fourier transform (FFT) with a Hamming window, and the average spectral power of each electrode at each frequency band was calculated by averaging the spectral powers of the nine-time segments. The asymmetry ratios between the right and left hemispheres were evaluated for each frequency band as (R-L)/(R + L), where R and L represent the spectral powers averaged over the right and left hemispheric electrodes, respectively. Consequently, 95 spectral power features (19 electrodes × 5 frequency bands) and 5 asymmetry ratio features (5 frequency bands) were evaluated for each trial. The LOOCV method was applied to assess the classification accuracy, considering the relatively small number of task trials performed. For each cross-validation, the sequential forward feature selection (SFFS) method was used to select the best feature subset for the current training dataset as well as to reduce the dimensionality of the feature vectors [38]. The 1-nearest neighbor error was used as the criterion function for the SFFS method, and the maximum number of selected features was set to 10 to prevent potential over-fitting of the data. LDA and support vector machine (SVM) classifiers were used to calculate the classification accuracies [39].

## Results

### Neurological assessment based on event-related potentials

**Fig. 3:**
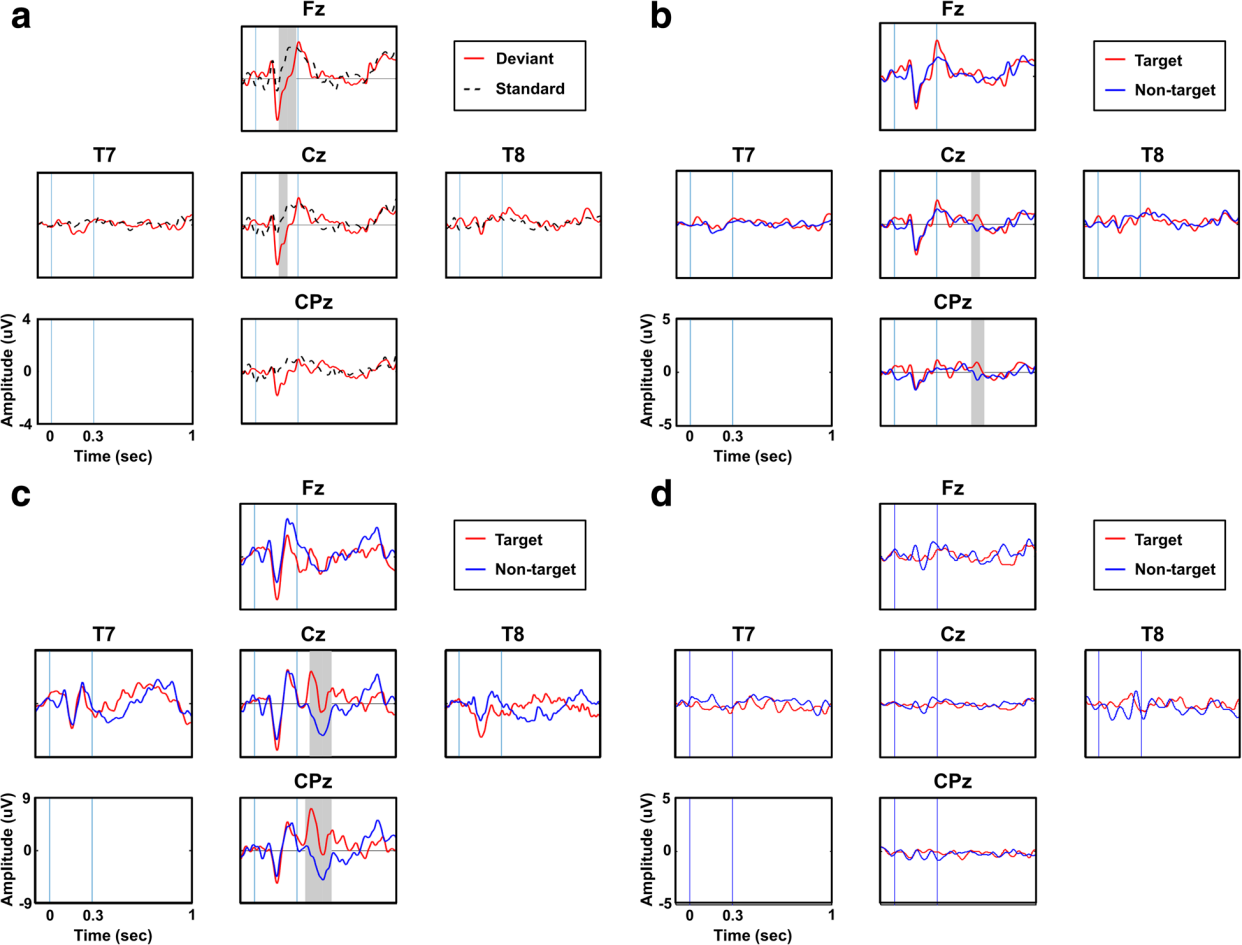
Results of the auditory oddball test. **a** Neural responses reflecting normal auditory function in the CLIS patient. Red bold and black dotted lines represent ERPs by deviant (240 trials) and standard (240 trials randomly selected from 600 trials) stimuli, respectively. The shaded area indicates the significant area as the result of the paired t-test between deviant and standard stimuli (*p*-value < 0.05). **b** Neural responses reflecting normal cognitive processes in the CLIS patient. Blue and red lines indicate ERPs by target (120 trials) and non-target (120 trials) stimuli, respectively. The shaded area indicates the significant area as the result of the paired t-test between target and non-target stimuli (*p*-value < 0.05). **c** An example of ERP waveforms obtained from a healthy subject after applying the same auditory oddball protocol used for the CLIS patient. The shaded area indicates the significant area as the result of the paired t-test between target and non-target stimuli (p-value < 0.05). **d** Neural responses showing abnormal cognitive decline after the CLIS patient’s physical status worsened. This result was obtained 4 months after the online experiment. We were unable to detect any evidence of consciousness or alertness of the patient

After IRB approval, neurological and associated signs found during the examined period between December 2015 to January 2016 confirmed that our participant had already progressed to CLIS based on the following evidence: there were no voluntary or involuntary eye movements, including blinking and corneal reflexes, with verbal commands or external stimuli such as light, tactile, and auditory stimuli. However, the participant’s pupillary light reflexes and cardiovascular response with stimulation were intact, and normal EEG alpha rhythms were observed. From these results, we could conclude that she was in CLIS at that time. After checking these neurological signs of CLIS, we evaluated her hearing functions and cognition using a neurological ERP test. We first checked for MMN after providing the participant with a series of frequent (standard) and rare (deviant) auditory stimuli [33]. Figure 3a shows the ERPs elicited by deviant and standard stimuli at five EEG channels, where distinct negative activity between 100 to 300 ms was observed in response to a deviant sound stimulus, with which we confirmed that the participant had normal hearing function. To ascertain the participant’s cognitive function, the patient was further instructed to selectively concentrate on specific beeps with high- or low-pitch tones. If the patient could not discriminate the beeps in accordance with our instructions, no difference would be observed in ERPs elicited by target and non-target stimuli. Figure 3b depicts the average ERP waveforms evoked by target and non-target stimuli, where significant differences were found in the amplitudes of two ERP waveforms at approximately 600 ms (the grey-coloured time period in Fig. 3b). When a healthy subject conducted the same auditory oddball paradigm, a P300 component was clearly observed at approximately 400 ms (see Fig. 3c). The prolonged latency and reduced amplitude of P300 might reflect the patient’s cognitive decline [40], but it seemed obvious that the patient retained sufficient cognitive function with which to understand the experimental instructions and perform basic mental tasks.

### ERD/ERS during each mental task

**Fig. 4:**
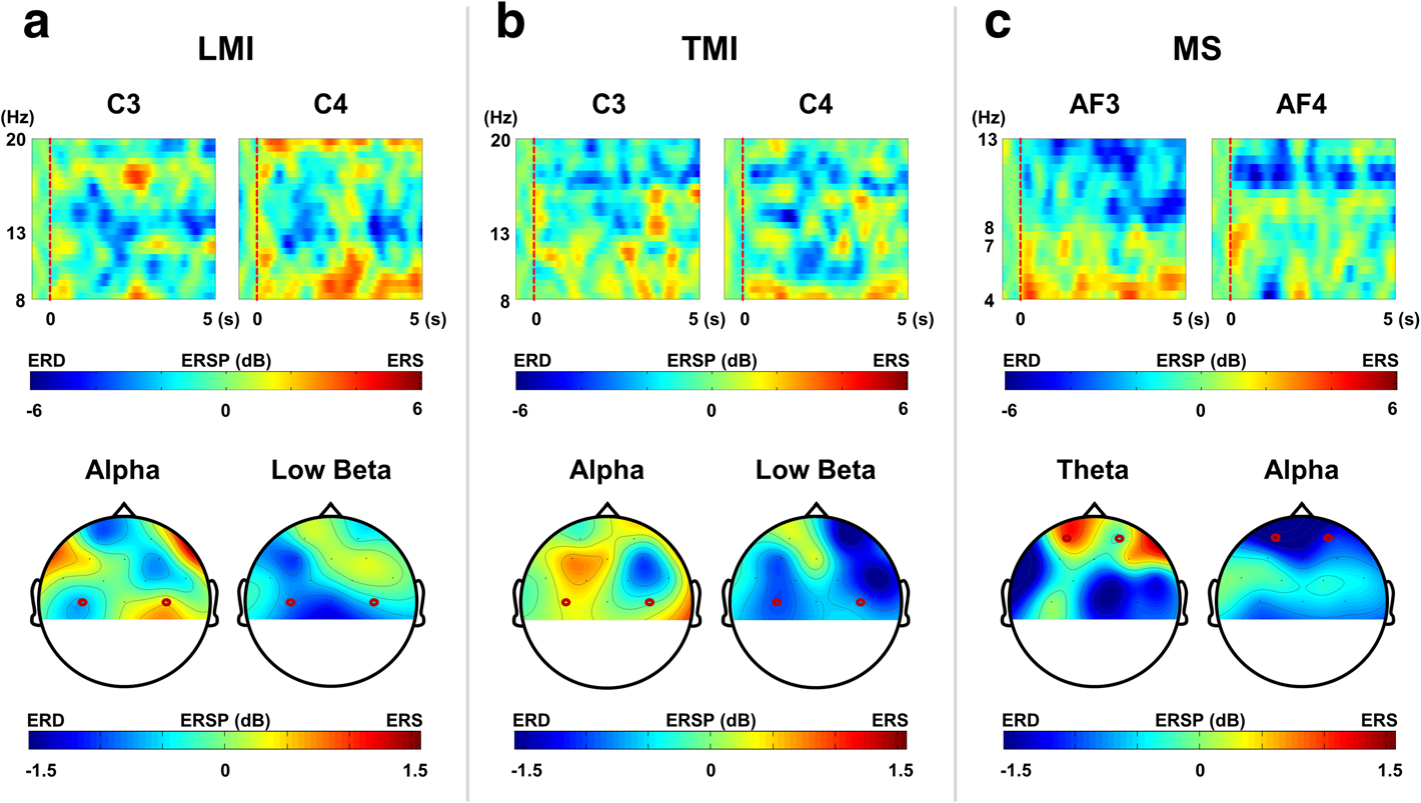
Time–frequency maps and topographical distribution for each mental task. **a** Event-related spectral perturbation (ERSP) maps during left-hand motor imagery (LMI) (**b**) ERSP maps during tongue motor imagery (TMI) (**c**) ERSP maps during mental subtraction (MS). EEG epochs from − 1 to 5 s were used for the time–frequency analysis. Red vertical dotted lines in the time–frequency maps of the first row represent the onset of each instruction. The values in the topographical distributions are averaged ERSP values from 0 to 5 s for each frequency band. The topographical maps in the posterior areas were not visualized because no electrode was attached in the parietal and occipital areas. Brown circles in the topographical maps of the second row indicate the electrodes used in the time–frequency analysis. All the figures were drawn using functions implemented in the EEGLAB toolbox (https://sccn.ucsd.edu/eeglab/index.php)

In the offline experiment, the patient was asked to perform one of three different mental tasks, namely LMI, MS, and TMI, the details of which can be found in the Methods section. We visualized ERS and ERD patterns during these mental tasks in order to confirm the subject’s engagement in the experiment. ERS and ERD represent stimulus/task-induced increase and decrease in spectral powers, respectively. The first row in Fig. 4 shows the ERSP maps at task-related channels for each task, and the second row illustrates the topographical maps of the averaged ERSP for specific frequency bands for each task. From our results, we could confirm that the characteristic ERS/ERD patterns were generated when the CLIS patient performed the given tasks. During the LMI task, we observed slight alpha ERS and ERD at C4 (contralateral side) and C3 (ipsilateral side), respectively. On the other hand, strong low-beta ERD was observed at both C3 and C4 (see Fig. 4a). During the TMI task, low-beta ERD patterns were commonly observed at both C3 and C4 (see Fig. 4b). Finally, the performance of the MS task enhanced the frontal theta powers and weakened the frontal alpha powers (see Fig. 4c). Distinct ERD/ERS patterns were observed especially between MS and motor-imagery tasks (LMI/TMI). These results are in line with previous studies [41–44], which show that LMI generates alpha ERD and beta ERD around the central areas of both hemispheres [41–44]. TMI generally generates beta ERS above the motor cortex; however, beta ERD above the left and right central areas is also often observed in prior studies [41, 45, 46]. Finally, several previous studies have reported that the mental arithmetic tasks enhance the frontal midline theta and weaken the frontal alpha [46–49], which is consistent with our findings.

### Results of the offline experiment

**Fig. 5:**
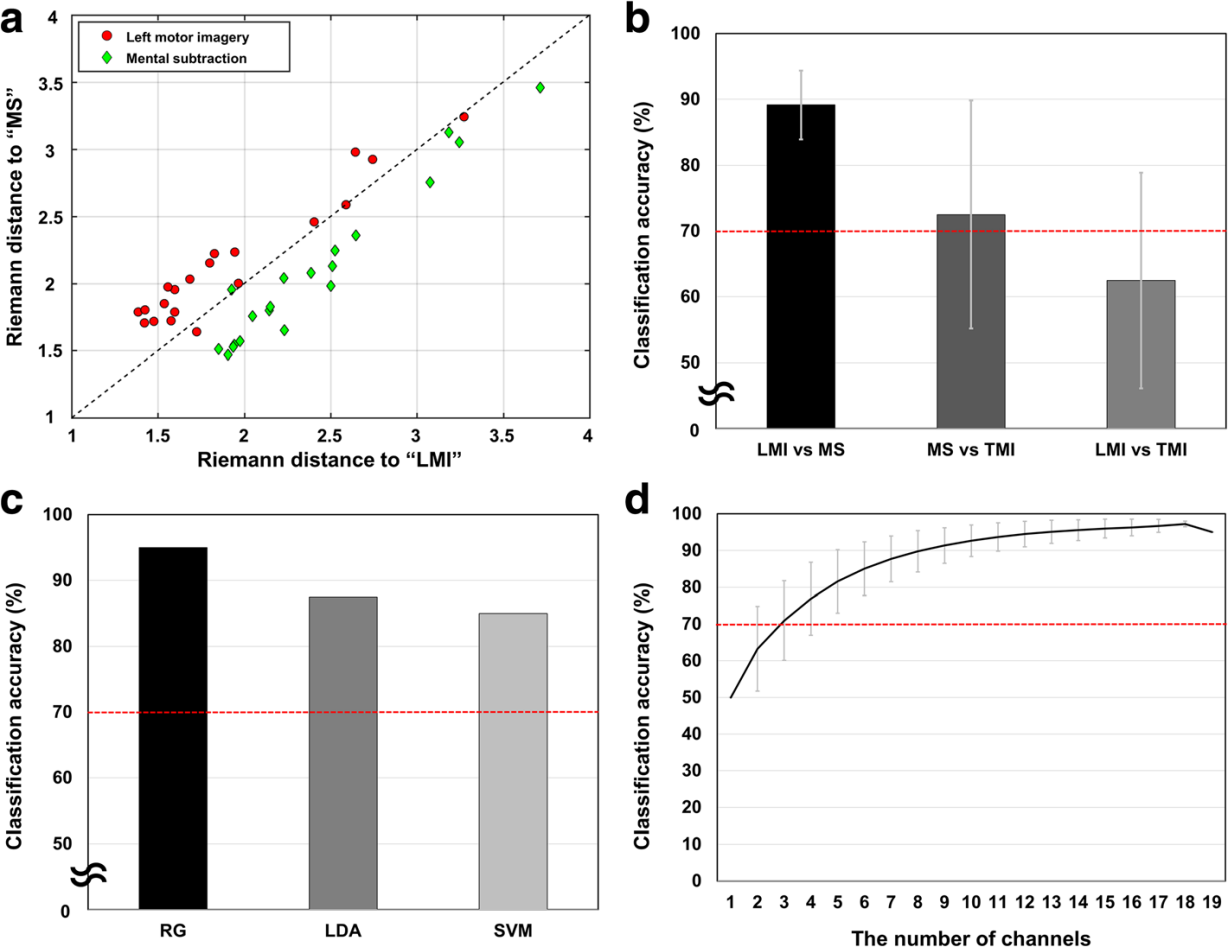
Results of the offline experiment. **a** Riemannian distance to the Riemannian mean of two class-related mean covariance matrices for LMI and MS. Each symbol represents a single 5-s trial and the dashed line indicates a decision border. This scatter plot was drawn after leave-one-out cross validation (LOOCV). **b** The average (cross-validated) classification accuracies of classifications for different combinations of three mental tasks: MS, LMI, and TMI. Nineteen electrodes were used for the classification. Each bar shows the average of the classification accuracies by different classification algorithms: Riemannian geometry (RG), linear discriminant analysis (LDA), and support vector machine (SVM). **c** The accuracies of classifying LMI and MS, evaluated using the three different classification methods. Nineteen electrodes were used. **d** The average offline classification accuracies with respect to the number of electrodes when RG was used for the classification. The classification accuracies were evaluated for all possible combinations of electrodes and then averaged

Through an offline experiment (Fig. 5), we determined the following factors: 1) the best combination of different mental tasks for binary communication and 2) the optimal classification method potentially leading to the highest performance. Figure 5a shows a manifold plot of Riemannian distance between LMI and MS, where each marker in the figure indicates the Riemannian distance of each trial from the two class-related mean covariance matrices, and the dashed line indicates the decision border. Most trials could be classified well even with the simplest linear classification scheme. Figure 5b shows the average cross-validated classification accuracies of binary classifications for different combinations of three mental tasks: MS, LMI, and TMI. Among the three possible combinations of mental tasks, the highest average classification accuracy of 89% was achieved when the LMI and MS pair was used. Figure 5c shows the accuracies of classifying LMI and MS evaluated using three different classification methods: RG, LDA, and SVM. An average classification accuracy of 95% was achieved when RG was used for the classification of LMI and MS. On the other hand, average classification accuracies of 87.5 and 85% were achieved when conventional LDA and SVM classifiers were used, respectively, which were much smaller than that of the RG method. To further investigate whether reasonable classification accuracy could be achieved even when fewer electrodes were used, we gradually reduced the number of electrodes used for RG-based classification and evaluated the resulting changes in the average classification accuracy. This information is important because using fewer EEG electrodes can reduce the time required to set up the EEG experiments. Figure 5d shows the average classification accuracies with respect to the number of electrodes. The classification accuracy increased as more electrodes were used but a fairly high classification accuracy exceeding 90% could be achieved with only ten electrodes. We also tested the feasibility of P300-based BCI using the ERP data recorded in the first experiment, but the performance of the mental-task-based BCI (accuracy = 90%; time for decision = 5 s) was much better than that of the P300-based BCI (accuracy = 80%; time for decision = 24 s).

### Online performance of our paradigm

**Fig. 6:**
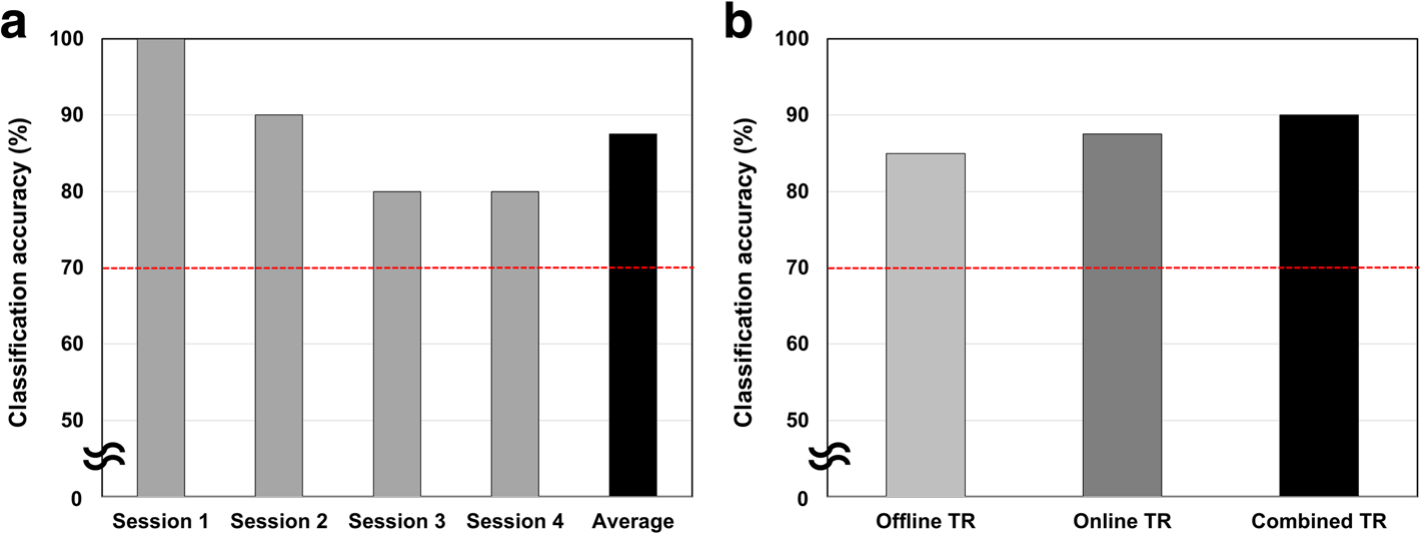
Results of the online experiment and test–retest reliability of the BCI system. **a** Results of the online experiment. RG was used as the classification algorithm. The number of electrodes was 19. The Y axis indicates the averaged online classification accuracies for each online run. The red horizontal dotted line indicates the level of chance. Note that the chance level is 70% when the number of trials is 20 and the confidence level is 99% [52] (**b**) Test–retest reliability of the BCI system. The Y axis indicates average classification accuracies for each training dataset

The results of our preliminary offline experiment were used to implement an online yes/no communication system. In the online experiment, we used the RG-based classification framework because it outperformed the traditional classification frameworks in the offline analysis. LMI and MS were selected as optimal tasks for the online experiments. Figure 6a shows the classification accuracy of the online experiment, the average accuracy of which was 87.5%. Although the online classification accuracy was slightly lower than the offline classification accuracy, our results are promising considering that no previous EEG-based BCI systems have achieved such a high accuracy with a patient in CLIS. The online classification accuracies for four consecutive runs were 100, 90, 80, and 80%. This gradual reduction in accuracy might be explained by the mental fatigue experienced by the patient, although the patient’s mental fatigue could not be quantitatively measured. A movie clip showing the online experiment can be found on YouTubeTM (https://youtu.be/zQWtSQOV50Q).

### Test-retest reliability

We evaluated the test–retest reliability of the proposed mental-imagery-based BCI paradigm. Figure 6b depicts the results of test–retest reliability assessments. In the online experiment, training datasets were recorded immediately before online experimental runs (denoted by Online TR), which resulted in an online classification accuracy of 87.5%, as previously shown in Fig. 6a. During the online experiments, EEG data were recorded and used to confirm the test–retest reliability. We trained the classifier using a training dataset recorded from the same patient about 6 months before the online experiment (denoted by Offline TR). This classifier, trained with the old training dataset, was also applied to the EEG data recorded during the online experiment. Even when the classifier was trained with the old training dataset, the classification accuracy reached 85%, demonstrating high test–retest reliability of our paradigm. In addition, a new dataset was constructed by combining the old training dataset (Offline TR) with a newly acquired training dataset (Online TR). By combining the two training datasets (denoted by Combined TR), the classification accuracy was further improved to 90%, as shown in Fig. 6b. Unfortunately, our patient was unable to participate in follow-up experiments. When we tested the patient four months after the online experiment, we could not find any evidence of consciousness or alertness (see Fig. 3d).

## Discussion

Our EEG-based BCI system showed good performance in terms of accuracy and communication speed. The offline and online average classification accuracies were 95 and 87.5%, respectively, and the time for each trial was just 5 s. Most previous EEG-based endogenous BCI methods have failed to effectively communicate with patients in CLIS [21, 27]. A study by De Massari et al. [21] proposed a Pavlovian semantic conditioning paradigm, which comprised the presentation of affirmative or negative statements for conditioned stimuli and electrical stimulation of skin for unconditioned stimuli, for the basic communication in CLIS. They reported a few cases with a classification accuracy of up to 70%, but the performance of their protocol was not consistent over all experimental runs. More recently, Chaudhary et al. [27] tested a binary paradigm for CLIS patients. Their paradigm had known answers and open questions. Their patients were automatically thought of ‘yes’ or ‘no’ for the answers to the questions. According to their reports, the EEG results did not exceed the chance-level threshold for correct communication. These previous EEG-based endogenous BCI studies have used paradigms based on passively generated automatic responses to the given questions. These passive paradigms have been used because the researchers assumed their CLIS patients might not perform active mental tasks due to cognitive decline or dementia originating from the neurodegenerative diseases. We carefully guess this might be a reason for the failure of previous EEG-based endogenous BCIs. If the patients in CLIS can perform active mental tasks such as LMI or MS, the performance of the EEG-based endogenous BCIs may be much improved because more robust EEG features can be extracted. Although previous EEG-based endogenous BCI studies have not yielded promising results for communication with patients in CLIS, recent NIRS-based BCI studies showed potential for use for communication with patients in CLIS [27, 50, 51]. For instance, Naito et al. proposed a NIRS-based BCI paradigm using mental tasks and tested its performance in a sample of 17 patients in CLIS [51]. About 40% of the patients in CLIS included in their study were able to successfully accomplish the experimental task with reliable performance, with an offline classification accuracy of approximately 80%. More recently, Gallegos-Ayala et al. proposed a new NIRS-based BCI paradigm that involved asking a patient in CLIS to think of either ‘yes’ or ‘no’ after yes-or-no questions [50], resulting in an average accuracy of 76.30%. An extended version of this NIRS study was reported in 2017 [27], in which the authors tested their NIRS-based BCI paradigm in a larger sample of patients in CLIS and confirmed that the patients could achieve an accuracy greater than 70%, better than that expected serendipitously. Although previous NIRS studies have shown promising results, the major disadvantages of these NIRS-based BCI paradigms are slow communication speeds and low classification accuracies. These paradigms generally require task lengths longer than 15 s for classification. In comparison, our method achieved both good classification accuracy (87.5%) and communication speed (5 s).

In 2017, a study by Guger et al. [26] demonstrated the potential of using a vibrotactile stimulation-based exogenous BCI paradigm for communication with patients in CLIS. However, the current study still makes an important contribution in the BCI community in that this is the first report showing that EEG-based ‘endogenous’ BCI paradigms can be used for online communication of patients in CLIS. Endogenous BCI is more ideal than exogenous BCI because it does not require any external stimuli in visual, auditory, and tactile forms, but only uses neural signals generated by performing designated mental-imagery tasks. In fact, the performance metrics of the present endogenous BCI were even better than those of the exogenous BCI applied to the patients in CLIS [26] (classification accuracy: 87.5% vs. 80% (two of three patients); communication speed: 5 s vs. 38 s). The only disadvantage of endogenous BCI paradigms might be the necessity of pre-training sessions to build classifiers (Note that the previous study [26] adopted an exogenous BCI paradigm, but it required training sessions); however, this study also showed that there were no significant differences in the classification accuracies even when a training dataset that was recorded several months prior to an online experiment was used for the online experiment. This suggests that patients in CLIS might use the proposed EEG-based BCI paradigm without any additional training runs for at least several months.

In 2012, Barachant et al. introduced a classification framework based on RG [35] that proved to be superior to conventional methods in terms of classification accuracy [35–37]. However, the performance of RG-based classification frameworks has never been evaluated for patients with ALS. In this study, we confirmed that the performance of an RG-based framework using covariance matrices as EEG descriptors outperformed LDA and SVM using spectral band powers as EEG features.

Brain signals may be the only feasible way for patients in CLIS to communicate with their external environment. In our study, a combination of LMI and MS showed the highest average classification accuracy of 89%, whereas the combination of LMI and TMI exhibited the lowest average classification accuracy of 62.5%. These results are in line with those of previous EEG-based BCI studies, in that the combination of motor and non-motor-imagery tasks resulted in better classification accuracy than the combination of two different motor-imagery tasks [30, 31].

We tested the proposed mental task-based BCI paradigm with only one patient in CLIS because it was extremely difficult to recruit patients who had lost all ability to communicate by conventional methods. Unfortunately, our patient was unable to participate in follow-up experiments after the only online experiment because her physical status worsened after recovering from acute pneumonia. When we revisited her and recorded her ERP signals while applying the auditory oddball paradigm, we were unable to find any evidence of consciousness or alertness (see Fig. 3d). We do not expect that our proposed BCI paradigm will be successfully applied to all patients in CLIS because every BCI paradigm suggested to date has failed in some participants. Nevertheless, we believe that our study is meaningful because our results suggest that EEG-based BCI systems can potentially be used for online binary communication with at least some patients in CLIS.

## Conclusions

In this study, we implemented an EEG-based BCI system that can be potentially used for online binary communication with a patient with ALS in CLIS. In the offline experiment, we determined the best combination of mental tasks and the optimal classification strategy leading to the best performance. In the online experiment, we investigated whether our BCI system could be potentially used for real-time communication with the patient. An online classification accuracy of 87.5% was achieved when RG-based classification was applied to real-time EEG data recorded while the patient was performing either left-hand motor imagery or mental subtraction task for 5 s. The offline and online results demonstrate that EEG-based endogenous BCI might be a feasible method for communication with patients who cannot communicate through conventional methods that require intact motor function.

## Abbreviations

ALS: Amyotrophic lateral sclerosis
BCI: Brain-computer interface
CAR: Common average reference
CLIS: Completely locked-in state
CMS: Common-mode-sense
DRL: Driven-right-leg
EEG: Electroencephalography
ERD: Event-related desynchronization
ERP: Event-related potential
ERS: Event-related synchronization
ERSP: Event-related spectral perturbation
FFT: Fast Fourier transform
fMRI: Functional magnetic-resonance imaging
ISI: Inter-stimulus interval
LDA: Linear discriminant analysis
LIS: Locked-in state
LMI: Left motor imagery
LOOCV: Leave-one-out cross validation
MEG: Magnetoencephalography
MI: Motor imagery
MMN: Mismatch negativity
MS: Mental subtraction
NIRS: Near-infrared spectroscopy
PSD: Power spectral density
RG: Riemannian geometry
SCM: Spatial covariance matrix
SFFS: Sequential forward feature selection
SSVEP: Steady-state visual evoked potential
SVM: Support vector machine
TMI: Tongue motor imagery
